# Autoimmune Diseases: Molecular Pathogenesis and Therapeutic Targets

**DOI:** 10.1002/mco2.70262

**Published:** 2025-06-16

**Authors:** Xiaoshuang Song, Hantian Liang, Fang Nan, Wenjing Chen, Junyao Li, Liu He, Yiping Cun, Zhenhong Li, Wei Zhang, Dunfang Zhang

**Affiliations:** ^1^ Department of Biotherapy, State Key Laboratory of Biotherapy and Cancer Center, Collaborative Innovation Center of Biotherapy West China Hospital Sichuan University Chengdu Sichuan China; ^2^ Center for Immunology and Hematology Department of Biotherapy and Cancer Center and State Key Laboratory of Biotherapy West China Hospital Sichuan University Chengdu Sichuan China; ^3^ Department of Immunology, Institute of Basic Medical Sciences, Chinese Academy of Medical Sciences; School of Basic Medicine Peking Union Medical College Beijing China

**Keywords:** autoimmune diseases, immune tolerance, pathogenesis, therapeutic strategies

## Abstract

Autoimmune diseases are a set of disorders in which the immune system attacks one's own tissues, leading to chronic inflammation, tissue damage, and systemic dysfunction. Affecting approximately 10% of the global population, these diseases impose significant health and economic burdens worldwide. The pathogenesis of autoimmune diseases is complex, involving not only genetic predisposition (e.g., human leukocyte antigen variants), environmental triggers (e.g., infections), and a dysregulated immune response but also various interacting components that contribute to the development of diverse clinical phenotypes. This review provides a comprehensive overview of common autoimmune diseases, covering their clinical manifestations, pathogenic mechanisms, and diagnostic approaches such as disease‐specific autoantibodies. We also explore current therapeutic strategies, including commonly used broad‐spectrum anti‐inflammatory drugs, recent molecular‐targeted therapies (e.g., Janus kinase inhibitors, monoclonal antibodies), and emerging cellular therapies such as chimeric antigen receptor T cells therapy and regulatory T‐cell adoptive transfer. Incorporating knowledge from preclinical and clinical studies, this review synthesizes relevant information to inform about autoimmune diseases, bridge the gap from lab to clinic, and promote future advances through exploring precision medicine applications to meet clinical needs.

## Graphical abstract

This review provides a comprehensive overview of common autoimmune diseases, details clinical manifestations and summarizes the pathogenesis, including the breakdown of immune tolerance, initiation of autoimmune responses, and their progressive amplification. We also explore current therapeutic strategies, including commonly used broad‐spectrum anti‐inflammatory drugs, recent molecular‐targeted therapies, and emerging cellular therapies, aiming to propel future research and therapeutic breakthroughs in this field.

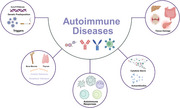

## 1 Introduction

Autoimmune diseases are a heterogeneous group of disorders characterized by the loss of immune self‐tolerance, leading to aberrant attacks on host tissues and organs. To date, over 100 distinct autoimmune diseases have been identified, with rheumatoid arthritis (RA), systemic lupus erythematosus (SLE), Sjögren's syndrome (SS), and systemic sclerosis (SSc) being the most prevalent [1]. Epidemiological studies estimate that autoimmune diseases collectively affect approximately 10% of the global population [2]. Notably, RA alone affected 17.6 million individuals worldwide in 2020, and this number is projected to rise by 80.2% to 31.7 million by 2050 [3]. Similarly, the global prevalence of SLE was recently estimated to be 3.41 million cases, with higher incidence rates in women and certain ethnic groups [4]. In addition to the negative affects patients’ quality of life, these systemic autoimmune disorders pose a substantial economic burden, highlighting the urgent need for the development of novel therapeutic strategies to improve disease management and treatment as well as patient outcomes [5].

The pathogenesis of autoimmune diseases is complex. Common mechanisms shared across these conditions primarily involve the breakdown of immune tolerance in T cells and B cells, along with abnormal cytokine production and pathway activation [6]. Given the intricate composition and function of the immune system, the progression of autoimmune diseases is difficult to predict. Current treatment approaches, focused primarily on immune suppression and symptom management, often do not achieve full disease control and are associated with significant side effects [7]. Therefore, in‐depth investigation into the precise mechanisms driving autoimmune responses is essential. Identifying novel therapeutic targets that could either restore immune tolerance or selectively modulate pathogenic immune pathways holds the promise of more effective treatments with fewer adverse effects.

In this review, we explore the key pathogenic mechanisms underlying autoimmunity, including loss of immune tolerance, dysregulated T‐ and B‐cell responses, and aberrant cytokine signaling. Additionally, we discuss current therapeutic strategies, ranging from conventional immunosuppression to targeted therapies, while highlighting emerging molecular targets and novel treatment approaches aimed at restoring immune homeostasis, which offer potential pathways toward more precise and effective therapies. By integrating recent advances in immunology and clinical research, this review aims to provide a comprehensive understanding of autoimmune pathogenesis and the evolving treatment landscape.

## 2 Classification and Clinical Manifestations of Autoimmune Diseases

**FIGURE 1:**
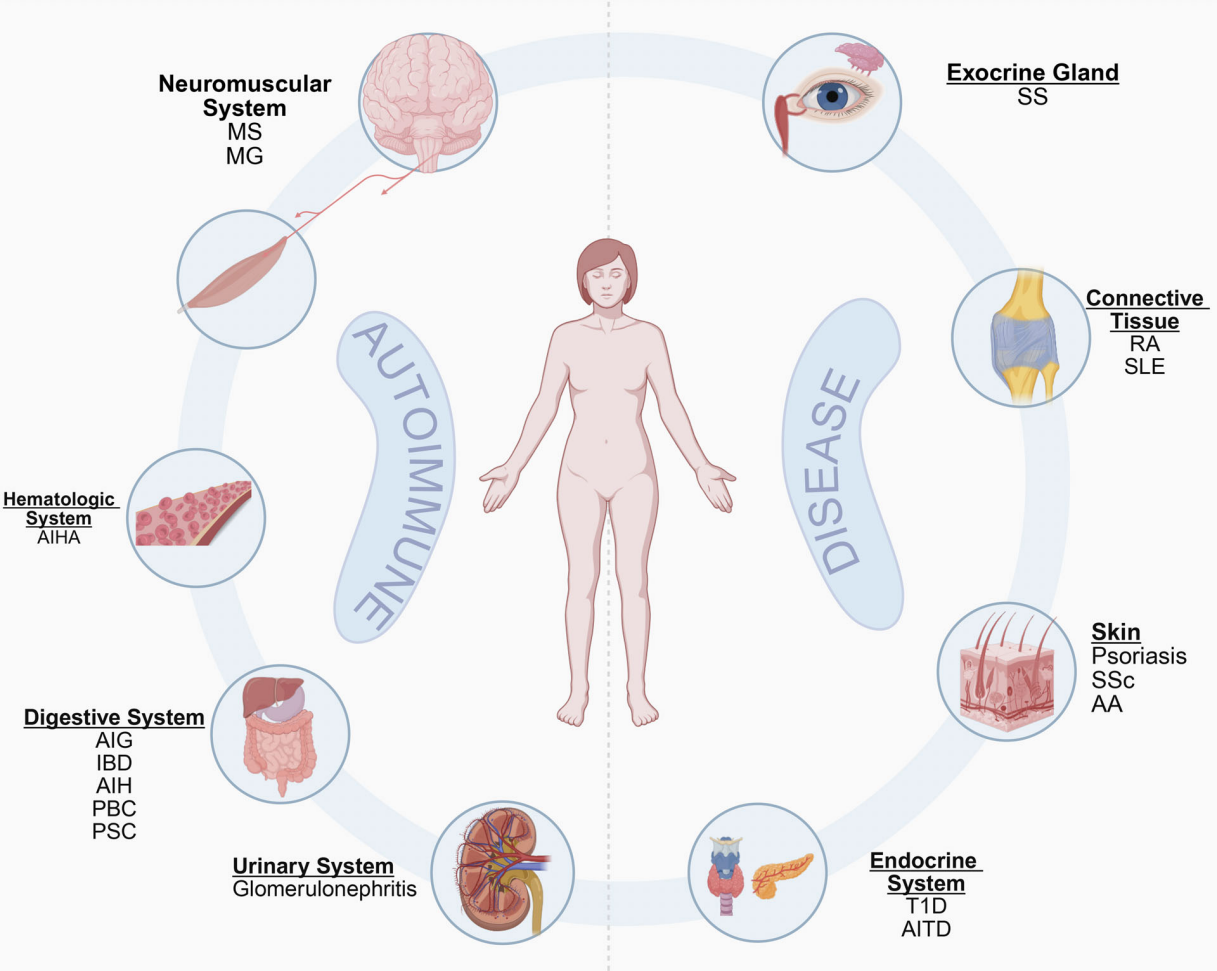
Common autoimmune diseases classified according to anatomical site. *Abbreviations*: AA, alopecia areata; AIG, autoimmune gastritis; AIH, autoimmune hepatitis; AIHA, autoimmune hemolytic anemia; AITD, autoimmune thyroid disease; RA, rheumatoid arthritis; SLE, systemic lupus erythematosus; SS, Sjögren's syndrome; SSc, systemic sclerosis; T1D, type 1 diabetes; IBD, inflammatory bowel disease; PBC, primary biliary cholangitis; PSC, primary sclerosing cholangitis; MS, multiple sclerosis; MG, myasthenia gravis.

Currently, autoimmune diseases lack a unified classification system but are commonly categorized based on the extent of tissue involvement into systemic and organ‐specific types. Systemic autoimmune diseases such as SLE and SS involve multiorgan damage due to widespread immune dysregulation, whereas organ‐specific variants such as Hashimoto's thyroiditis (HT) and myasthenia gravis (MG) predominantly affect specific organs or glands. Clinically, these disorders are also stratified by their primary anatomical targets, such as connective tissue (e.g., RA and SLE), exocrine glands (e.g., SS), skin (e.g., psoriasis), the digestive system (e.g., ulcerative colitis and autoimmune gastritis [AIG]), the endocrine system (e.g., type 1 diabetes [T1D] and chronic thyroiditis), the hematologic system (e.g., autoimmune hemolytic anemia [AIHA]), the neuromuscular system (e.g., multiple sclerosis [MS]), and the urinary system (e.g., glomerulonephritis). Although this review uses an anatomical classification framework to systematically present clinical manifestations of autoimmune diseases (Figure 1), many autoimmune diseases are not confined to a single anatomical site due to their characteristic multitissue involvement. Representative examples include SLE and SSc, which typically demonstrate multisystem pathology affecting diverse organ systems.

### 2.1 Connective Tissue

RA is a chronic systemic inflammatory disease characterized pathologically by symmetrical and persistent polyarticular pain, accompanied by chronic inflammation of the synovial membranes and the formation of vascular pannus [8]. RA leads to cartilage and bone destruction as well as joint inflammation, leading to joint deformities and functional loss. Moreover, systemic inflammation may cause damage to other organs, and have a negative effect on patients’ quality of life [9, 10].

SLE is a heterogeneous systemic autoimmune disease characterized by a wide variety of clinical manifestations, complicating both its assessment and treatment [11]. Patients with SLE produce large amounts of autoantibodies, potentially affecting multiple organs and systems [12]. According to the clinical assessment and diagnostic criteria established by the Systemic Lupus International Collaborating Clinics, the clinical manifestations of SLE can include acute cutaneous lupus, chronic cutaneous lupus, oral or nasal ulcers, nonscarring alopecia, arthritis, serositis, nephritis, hemolytic anemia (HA), and neurological disorders such as seizures [13, 14].

### 2.2 Exocrine Glands

SS is a chronic disease that primarily affects the exocrine glands, particularly the salivary and lacrimal glands. In addition, the nasal cavity, upper respiratory tract, and female genitalia may be affected. Clinically, the initial symptoms manifest as dryness of the mouth and eyes. Owing to decreased saliva production, patients experience dysphagia, altered taste sensations, oral burning, difficulty speaking continuously, and an urge to wake up at night to drink water. Individuals with SS have an increased incidence of oral infections, gingivitis, and dental caries and often report symptoms such as dryness, itching, pain, and photophobia in the eyes, which are prone to infection. Additionally, parotid gland swelling is common among affected individuals. In addition to affecting the glands, SS affects the skin, joints, lungs, kidneys, muscles, and nerves. This can result in a range of clinical manifestations, including vasculitis, Raynaud's phenomenon, arthritis, arthralgia, interstitial pneumonia, tracheobronchial dryness, interstitial nephritis, lymphoma, myalgia, neuroinflammation, and hemiplegia [15, 16, 17].

### 2.3 Skin

Psoriasis, commonly referred to as psoriasis vulgaris, is a chronic inflammatory skin condition [18]. It is classified into five distinct clinical subtypes: plaque, guttate, inverse, pustular, and erythrodermic. Plaque psoriasis, the most prevalent subtype, is characterized by well‐defined, red, itchy plaques on the trunk, limbs, and scalp, with multiple layers of silvery‐white scales. Guttate psoriasis presents as small, acute erythematous spots, typically triggered by infections with Group A beta‐hemolytic *Streptococcus* and predominantly affects children and adolescents. Approximately one‐third of patients with guttate psoriasis subsequently develop plaque psoriasis [19, 20]. Inverse psoriasis affects intertriginous areas such as the groin, vulva, and axillae, resulting in mildly eroded erythema. Pustular psoriasis is distinguished by the presence of dense, sterile pustules on the skin surface [21, 22]. Erythrodermic psoriasis, the rarest type of psoriasis, often arises from prolonged use glucocorticoids and is associated with severe symptoms, including widespread erythema [23]. Psoriasis is associated with several comorbidities, including psoriatic arthritis, obesity, and metabolic syndrome [24].

SSc is the most fatal rheumatic disease. It is classified into several subtypes, including diffuse scleroderma; limited scleroderma; scleroderma without skin involvement; overlap syndromes (which occur simultaneously with other rheumatologic diseases); and calcinosis, Raynaud's phenomenon, esophageal dysmotility, sclerodactyly, and telangiectasias (CREST) syndrome [25, 26]. Early symptoms include skin tightness, itching, Raynaud's phenomenon, arthritis, and muscle pain. Skin lesions progress through three phases: the inflammatory, sclerotic, and atrophic phases. In patients with SSc, the digestive system is the most commonly affected organ system. Patients frequently present with symptoms such as reflux, bloating, constipation, diarrhea, and incontinence. Additionally, pulmonary fibrosis is common in patients with SSc [27]. Other organs, including the heart, kidneys, and thyroid, are also affected in SSc.

Alopecia areata (AA) is a temporary, nonscarring form of hair loss without permanent damage to hair follicles. AA is clinically characterized by round or oval patches of sudden hair loss, typically located on the scalp. This condition occurs when the immune system targets the hair follicles, resulting in inflammation [28].

### 2.4 Digestive System

Autoimmune liver disease refers to a group of chronic liver conditions caused by disturbances in the body's immune function. This includes autoimmune hepatitis (AIH), primary biliary cholangitis (PBC), primary sclerosing cholangitis (PSC), and overlap syndromes. Each autoimmune liver disease type presents with distinct clinical manifestations and varying degrees of pathological damage to the liver. AIH is characterized by symptoms such as fatigue, nausea, abdominal pain, and loss of appetite, accompanied by significant jaundice. AIH can progress and may lead to cirrhosis and liver failure [29]. It frequently has an insidious onset, with patients remaining asymptomatic for extended periods. Patients with acute manifestations can develop acute liver failure [29]. PBC is a chronic cholestatic liver disease that presents with nonsuppurative injury to the small bile ducts within the liver and damage to the portal areas. In some cases, patients present with nonspecific symptoms, such as fatigue and itchy skin. PBC can progress to portal hypertension, liver fibrosis, and cirrhosis [30]. PSC causes inflammation and fibrosis of both intrahepatic and extrahepatic bile ducts, leading to bile duct strictures and cholestasis. In addition, it is associated with inflammatory bowel disease (IBD). In later stages, patients with PSC present with signs of decompensated cirrhosis, including portal hypertension and liver failure. The risk of PSC recurrence is high, even after liver transplantation. Additionally, patients with PSC have a higher risk of developing cholangiocarcinoma and colorectal cancer [31, 32].

AIG is a chronic inflammatory disease that leads to gastric dysfunction, vitamin B12 and iron malabsorption, and pernicious anemia (which is responsible for the primary symptoms of AIG) [33]. Furthermore, vitamin B12 deficiency affects the gastrointestinal and nervous systems, resulting in symptoms such as diarrhea, glossitis, peripheral neuropathy, weakness, memory loss, and confusion [34]. The lack of gastric acid also contributes to delayed gastric emptying, increasing the risk of gastrointestinal infections [35, 36]. In later stages, AIG is characterized by gastric mucosal atrophy and an elevated risk of gastric neoplasms [37].

IBD is an autoimmune‐related idiopathic intestinal inflammatory disorder, clinically characterized by symptoms such as abdominal pain, diarrhea, bloody stools, and weight loss. IBD primarily affects the ileum, rectum, and colon. The main forms are ulcerative colitis and Crohn's disease [38, 39]. Ulcerative colitis involves the rectum, with inflammation limited to the mucosa and submucosa, often accompanied by crypt inflammation and crypt abscesses. In contrast, Crohn's disease can occur in any part of the digestive system; however, it most commonly affects the terminal ileum. It is characterized histologically by thickening of the submucosa, transmural inflammation, fissuring ulcers, and granulomas. Complications of IBD include fibrosis‐induced intestinal strictures and obstruction as well as an increased risk of colorectal cancer [40, 41, 42]. Furthermore, patients with IBD experience various extraintestinal manifestations including ocular conditions such as uveitis and scleritis; skin and mucosal lesions such as erythema nodosum and pyoderma gangrenosum; arthritis; low bone mass and osteoporosis; liver and pancreaticobiliary disorders such as PSC, nonalcoholic fatty liver disease, and acute pancreatitis; and portal vein thrombosis [43, 44].

### 2.5 Endocrine System

T1D, characterized by the destruction of pancreatic β‐cells and insufficient insulin production, primarily affects children and adolescents [45, 46]. Delayed treatment or inadequate disease management may hinder children's development. Patients have high blood glucose levels, and symptoms such as polyuria, polydipsia, polyphagia, and weight loss [47]. Acute T1D complications include diabetic ketoacidosis, hypoglycemia, and infections. Additionally, the improper use of insulin injections may lead to chronic complications such as joint disorders, cataracts, and osteoporosis. Chronic complications such as macrovascular and microvascular diseases, as well as neuropathy, are significant contributors to diabetes‐related disability and mortality [48].

Autoimmune thyroid disease (AITD) refers to a group of disorders in which the immune system attacks thyroid antigens, leading to lymphocytic infiltration of the thyroid tissue. The most common forms of AITD are Graves’ disease (GD) and HT [49]. GD, also known as toxic diffuse goiter, is characterized by hyperthyroidism caused by abnormal antibodies that activate thyroid‐stimulating hormone receptors, resulting in thyroid hyperplasia. However, a small percentage of patients with GD present with thyroid dysfunction [50]. Symptoms associated with GD include hyperthyroidism, mucinous edema of the skin, reproductive system abnormalities, and Graves’ orbitopathy [51]. The latter is an important extrathyroidal manifestation of GD, in which patients experience prominent exophthalmos, photophobia, pain, and conjunctival or corneal lesions [52]. In contrast, in HT, the immune system attacks thyroid follicular cells, leading to hypothyroidism. Clinically, HT presents as an enlarged, firm, and grayish thyroid gland that compresses the neck, resulting in difficulties with speech, breathing, and swallowing. Additional symptoms include constipation; pseudo‐obstruction; intestinal obstruction; dry, yellowed, and thickened skin; muscle pain and spasms; and anemia [53].

### 2.6 Hematologic System

AIHA is a type of HA caused by immune system dysfunction, in which antibodies and/or complement against self‐red blood cells are produced and bind to red blood cell membrane antigens, accelerating the destruction of red blood cells. It is mostly chronic extravascular hemolysis, with a slow onset and more common in adult women. It is characterized by anemia, jaundice, and splenomegaly. One‐third of the patients have anemia and jaundice, more than half have mild to moderate splenomegaly, and one‐third have hepatomegaly [54].

### 2.7 Neuromuscular System

MS is an autoimmune disease primarily characterized by inflammatory demyelinating lesions in the white matter of the central nervous system [55]. Patients with MS develop demyelinating plaques within the periventricular white matter, optic nerves, spinal cord, brainstem, and cerebellum [56]. Clinically, MS presents as muscle weakness, optic neuritis, nystagmus, sensory disturbances, and bladder dysfunction [57, 58]. Based on disease progression, the National Multiple Sclerosis Society (USA) classifies MS into four types: relapsing‐remitting (RR), secondary progressive (SP), primary progressive (PP), and progressive‐relapsing (PR) [59]. The RR type is characterized by relapses in the early stages, followed by periods of remission. Over time, many patients with long‐standing RR transition to the SP type, in which the condition worsens without remission. The PP type manifests in middle to late adulthood, leading to a gradual decline in function. The PR type is relatively rare and is similar to PP but with acute relapses.

MG is an autoimmune disease characterized by dysfunction in transmission at the neuromuscular junction. This occurs when antibodies bind to acetylcholine receptors or other functionally related molecules on the postsynaptic membrane, disrupting normal signal transmission and leading to partial or generalized skeletal muscle weakness and easy fatigue [60]. The primary muscles that are weakened in MG include the extraocular, bulbar, limb, and axial muscles. Approximately 60% of affected individuals initially present with symptoms such as ptosis or diplopia, which may progress to ocular MG over time [61]. In addition, patients with MG experience swallowing and chewing difficulties, limb weakness, and respiratory distress [62]. In the early stages of MG, the majority of patients have thymic hyperplasia, which can progress to thymic atrophy or thymoma. Therefore, thymectomy is commonly performed to alleviate symptoms in patients with MG [63]. Furthermore, approximately 15% of patients with MG develop comorbidities such as thyroiditis and SLE [64].

### 2.8 Urinary System

Glomerulonephritis is a heterogeneous group of diseases characterized by clinical features such as hematuria, proteinuria, hypertension, edema, oliguria, and varying degrees of renal dysfunction [65]. Pathologically, it is marked by inflammation of the glomeruli and hypercellularity, which can lead to secondary kidney damage [65]. Based on different pathogenic mechanisms, glomerulonephritis is broadly classified into several categories: immune complex‐mediated glomerulonephritis, antineutrophil cytoplasmic antibody‐associated glomerulonephritis, antiglomerular basement membrane glomerulonephritis, C3 glomerulopathy, and monoclonal immunoglobulin‐related glomerulonephritis [66, 67, 68]. With the exception of monoclonal immunoglobulin‐related glomerulonephritis, all glomerulonephritis subtypes are associated with varying degrees of autoimmune dysregulation. Glomerulonephritis can also be classified based on the clinical type [69]. Clinically, the most common form of glomerulonephritis is acute glomerulonephritis, caused by streptococcal infection, with the incidence being highest in children living in regions and countries with poor sanitary conditions [70]. Glomerulonephritis‐related complications include severe congestion, heart failure, hypertensive encephalopathy, and acute renal failure.

## 3 Pathogenesis of Autoimmune Diseases

Autoimmune diseases arise from the aberrant recognition and attack of self‐antigens by the immune system, rooted in the breakdown of immune tolerance, a physiological safeguard governed by central and peripheral tolerance mechanisms. Under healthy conditions, the immune system distinguishes “self” from “non‐self” through a multilayered regulatory process that includes clonal deletion of self‐reactive lymphocytes in the thymus and bone marrow, and suppression by regulatory T cells (Tregs) within peripheral tissues [71]. However, the convergence of genetic predisposition, environmental triggers, and immune‐regulatory imbalances creates a “tripartite assault” that dismantles these tolerance mechanisms. This enables the escape of autoreactive T‐ and B‐cells, activation of inflammatory cascades, and irreversible tissue damage, which drives the pathogenesis of autoimmune diseases [72].

### 3.1 Basic Composition and Functions of the Immune System

The immune system is a sophisticated and dynamic network of cells, tissues, and molecules that collectively safeguard the body against infection, eliminate abnormal cells, and maintain internal stability [73]. Composed of innate and adaptive immune components, it operates through specialized cell development, activation, and function. The innate immune system provides rapid, nonspecific defense mechanisms, whereas the adaptive immune system offers precise, long‐lasting immunity through antigen‐specific responses [74]. Together, these systems perform three critical functions: immune defense, which protects against external pathogens; immune surveillance, which identifies and eliminates abnormal cells such as tumors; and immune homeostasis, which ensures the balance and stability of the internal environment. This intricate interplay of components and functions highlights the essential role of the immune system in maintaining health and preventing disease.

#### 3.1.1 Basic Composition of the Immune System

**FIGURE 2:**
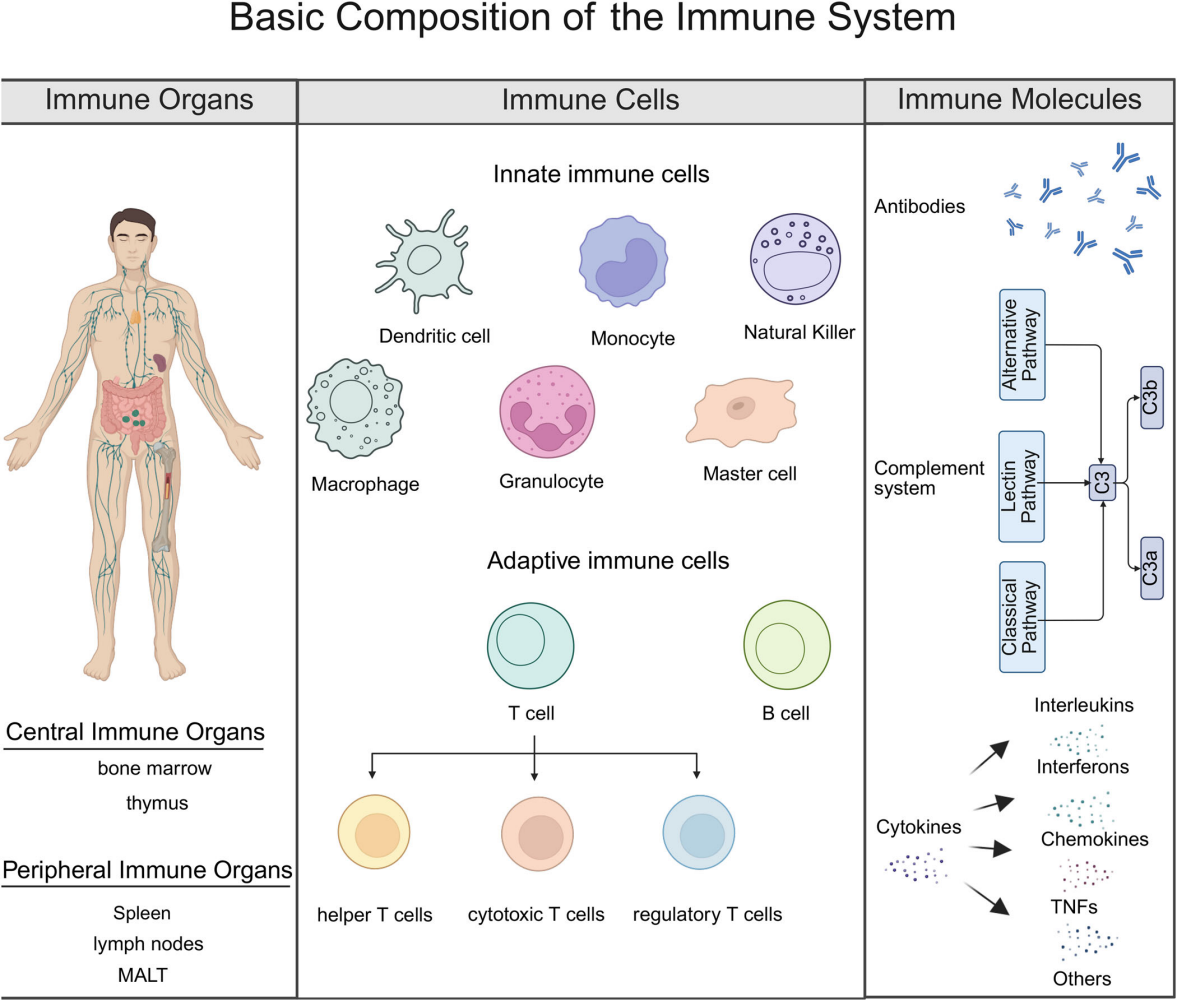
Components of the immune system. The immune system comprises immune organs (central: bone marrow, thymus; peripheral: lymph nodes, spleen, mucosa‐associated lymphoid tissue), immune cells (innate: phagocytes, NK cells; adaptive: T and B lymphocytes), and immune molecules (antibodies, complement system, cytokines). These components collectively mediate immune defense, surveillance, and homeostasis. Dysregulation in any component can contribute to the pathogenesis of autoimmune disease.

The immune system is composed of immune organs, immune cells, and immune molecules, which work together to maintain immune homeostasis and defend the body against pathogens (Figure 2).

##### 3.1.1.1 Immune Organs

Immune organs are sites in which immune cells are generated, develop, mature, and function. They are divided into central and peripheral immune organs [75].

Central immune organs include the bone marrow and thymus. The bone marrow is the site in which hematopoietic stem cells differentiate into various blood cells and immune cells, and B lymphocytes mature. The thymus is the central site for the differentiation, development, and maturation of T lymphocytes. Lymphoid stem cells migrate to the thymus, undergo a complex selection process, and differentiate into immunocompetent T lymphocytes.

Peripheral immune organs primarily include lymph nodes, the spleen, and mucosa‐associated lymphoid tissue (MALT). Lymph nodes filter lymph and remove pathogens, serving as important sites for housing lymphocytes and immune responses. The spleen filters blood, clears aged red blood cells and pathogens, and activates immune cells to initiate immune responses. MALT, distributed in mucosal areas such as the respiratory and digestive tracts, forms the first line of defense against pathogen invasion.

##### 3.1.1.2 Immune Cells

Immune cells carry out the work of the immune system and can be divided into innate and adaptive immune cells. Innate immune cells include phagocytes (such as neutrophils and macrophages), natural killer (NK) cells, and dendritic cells (DCs). Phagocytes eliminate pathogens through phagocytosis, whereas DCs are antigen‐presenting cells that activate naïve T lymphocytes and initiate adaptive immune responses. NK cells directly kill virus‐infected cells and tumor cells, playing a crucial role in immune surveillance [76]. Adaptive immune cells consist mainly of T and B lymphocytes. T lymphocytes can be further divided into helper T cells, cytotoxic T cells (CTL), and Tregs, which are responsible for immune regulation, target‐cell killing, and maintaining immune homeostasis, respectively. B lymphocytes differentiate into plasma cells on antigen stimulation, producing antibodies that mediate humoral immunity [77].

##### 3.1.1.3 Immune Molecules

Immune molecules are essential components of the immune system, assisting immune cells in carrying out immune responses. They include antibodies, the complement system, and cytokines.

Antibodies, produced by B lymphocytes, are Y‐shaped proteins critical to adaptive immunity. Their variable regions enable precise antigen recognition, neutralizing pathogens through toxin blockade and inhibiting viral entry. Simultaneously, the constant (Fc) region mediates effector functions: opsonizing pathogens for phagocyte clearance, activating the complement cascade, and forming immune complexes for neutrophil recruitment. This dual targeting (antigen binding and immune activation) ensures coordinated pathogen elimination [78].

The complement system, activated through three distinct pathways (classic via antigen–antibody complexes, alternative via spontaneous hydrolysis, and lectin via pathogen carbohydrate recognition), executes a multifaceted immune defense. In addition to directly lysing pathogens through membrane attack‐complex formation, it enhances phagocytosis by opsonizing microbes with C3b fragments and amplifies inflammatory responses via anaphylatoxins (C3a, C5a) that recruit leukocytes. Additionally, complement coordinates with adaptive immunity by solubilizing immune complexes and priming B‐cell responses, while regulatory proteins prevent host tissue damage through controlled activation cascades [79].

Cytokines such as interleukins (ILs), interferons (IFNs), tumor necrosis factors (TNFs), and chemokines, act as molecular messengers orchestrating immune dynamics. Secreted by immune cells (e.g., T cells, macrophages) and stromal cells, they regulate immune cell activation, proliferation, and differentiation via autocrine, paracrine, or endocrine signaling. For example, IFNs prime antiviral defenses by inducing antiviral protein synthesis, whereas TNF‐α drives inflammation and leukocyte recruitment. Additionally, cytokines coordinate innate and adaptive immunity by modulating antigen presentation (IL‐12) and antibody class‐switching (IL‐4 and IL‐6). Their pleiotropic effects, mediated through janus kinase (JAK)–signal transducer and activator of transcription (STAT) or nuclear factor kappa‐B (NF‐κB) pathways, balance proinflammatory and anti‐inflammatory responses, ensuring targeted pathogen clearance while preventing immunopathology [80].

#### 3.1.2 Functions of the Immune System

The immune system performs three primary functions: immune defense, immune surveillance, and immune homeostasis. These functions collectively protect the body from external threats, eliminate internal abnormalities, and maintain internal stability.

##### 3.1.2.1 Immune Defense

Immune defense, aimed at protecting the body from invading pathogens such as bacteria, viruses, fungi, and other microorganisms, is the primary function of the immune system [81]. The immune system rapidly recognizes and responds to these threats through a multilayered defense mechanism. Physical barriers, such as the skin and mucous membranes, serve as the first line of defense, preventing the entry of most pathogens. When pathogens breach these barriers, immune cells and molecules are activated to neutralize the threat. For example, phagocytes such as macrophages engulf and digest pathogens, whereas lymphocytes produce specific antibodies or kill infected cells directly. These coordinated actions effectively eliminate pathogens, preventing the establishment and spread of infections [82].

##### 3.1.2.2 Immune Surveillance

The immune surveillance function of the immune system involves the identification and elimination of abnormal cells, including tumor cells and virus‐infected cells [83, 84, 85]. During cell growth and division, genetic mutations or exposure to mutagenic substances can lead to cellular abnormalities. Under normal conditions, the immune system continuously monitors cellular states by recognizing cell surface antigens, enabling the timely detection and elimination of abnormal cells. For example, T cells can specifically recognize tumor‐specific antigen peptides on the surface of tumor cells presented by major histocompatibility complex (MHC) molecules, leading to the direct killing of these cells. This process helps suppress tumor development in the early stage, thereby maintaining overall health [86].

##### 3.1.2.3 Immune Homeostasis

Immune homeostasis ensures the stability of the internal environment. In normal physiological processes, cells undergo constant turnover, resulting in the production of aged, damaged, or dead cells. The immune system identifies and clears these cells, as well as metabolic waste and toxic substances [87]. Additionally, the immune system must precisely regulate the intensity of immune responses to avoid excessive reactions that could damage healthy tissues [88]. This delicate balance is crucial for maintaining internal stability and preventing autoimmune disorders.

### 3.2 Immune Tolerance

The immune system is dedicated to detecting and eliminating foreign antigens while coordinating both proinflammatory and anti‐inflammatory responses. This dynamic equilibrium is known as immune homeostasis, a state essential for maintaining overall health [89]. The disruption of immune homeostasis can lead to infections and tumor development due to impaired immune responses (immunodeficiency), whereas excessive immune responses may result in autoimmune disorders. Despite the presence of self‐reactive T and B cells in healthy individuals, lymphocytes coordinate with other immune cells through a tightly regulated network to suppress autoimmunity and maintain tissue integrity. The balance of the immune system is maintained through central and peripheral tolerance mechanisms targeting T and B cells. Central tolerance eliminates potentially autoreactive T cells through negative selection in primary lymphoid organs, the bone marrow, and thymus [90]. Despite the stringency of central tolerance, significant numbers of autoreactive lymphocytes persist in the circulation of healthy individuals and retain the capacity to trigger autoimmunity [91]. Unwanted peripheral immune responses are suppressed through Treg‐mediated deletion and induction of anergy, mechanisms that constitute peripheral tolerance [92]. The mechanisms underlying immune tolerance encompass clonal deletion, anergy, immune regulation, and ignoring them. Therefore, the integrated mechanisms of central and peripheral tolerance establish and sustain immune homeostasis.

#### 3.2.1 Central Tolerance

##### 3.2.1.1 T‐Cell Tolerance

**FIGURE 3:**
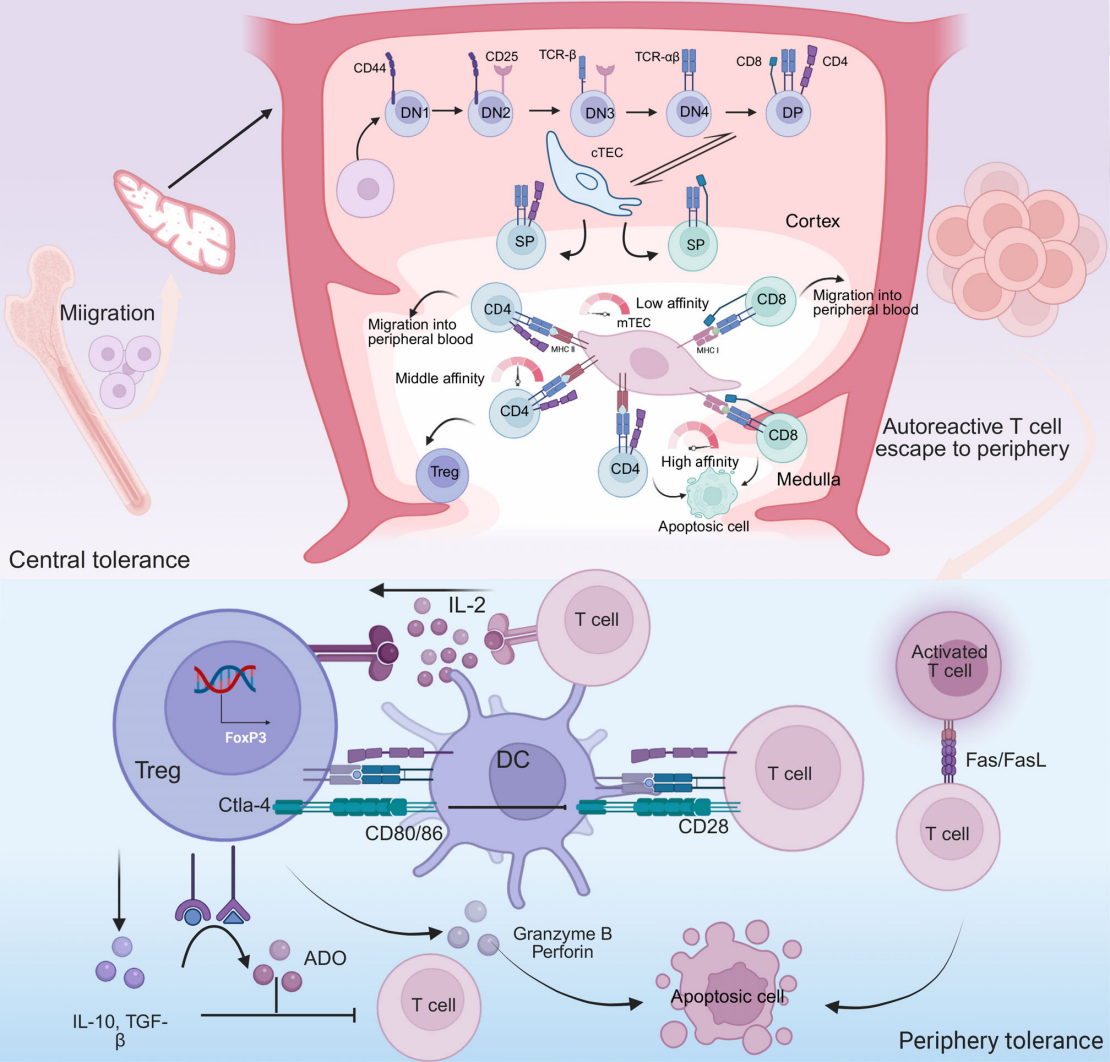
The mechanism of T‐cell tolerance. Central tolerance is established in the thymus. Bone marrow‐derived progenitor cells mature into DP thymocytes within the thymic cortex, where thymic cortical epithelial cells (cTECs) regulate the positive selection of DP thymocytes. Following positive selection, single‐positive (SP) thymocytes migrate to the thymic medulla. There, they undergo further screening based on their affinity for self‐peptides presented by medullary thymic epithelial cells (mTECs). SP thymocytes exhibiting low affinity for self‐antigens are permitted to egress into the peripheral circulation or lymphoid tissues. Those with intermediate affinity may differentiate into regulatory T (Treg) cells, while the remains displaying high affinity for self‐peptides are eliminated via apoptosis. Impairment of negative selection in the thymic medulla can lead to the escape of autoreactive T‐cell clones into the periphery. The activation of autoreactive T cells and excessive T‐cell‐mediated immune response are limited by the FasL interaction. Treg cells exert immunosuppressive effects through multiple mechanisms, including the secretion of inhibitory cytokines (IL‐10, TGF‐β), cytotoxic molecules (granzyme B, perforin), and adenosine (ADO), as well as the expression of immune checkpoint molecules such as cytotoxic T lymphocyte associated protein‐4 (CTLA‐4) and the high‐affinity interleukin‐2 receptor α‐chain (CD25).

Central tolerance of T cells occurs in the thymus, effectively preventing the escape of self‐reactive cells to the periphery and reducing the tendency toward autoimmunity. Bone marrow‐derived progenitors migrate to the thymus, where T‐cell antigen receptor (TCR) gene rearrangement at the CD4 and CD8 double‐negative stage generates either αβ or γδ progenitors with a diverse TCR repertoire capable of recognizing various antigens. Some αβ progenitors develop into CD4 and CD8 double‐positive (DP) thymocytes while acquiring functional TCR expression. DP thymocytes that exhibit high affinity for peptide–MHC complexes on thymic cortical epithelial cells (cTECs) receive survival signals and differentiate into either CD4+ or CD8+ single‐positive T cells during positive selection (Figure 3). Those that fail to interact with peptide–MHC complexes undergo apoptosis [93]. This process endows CD4+ or CD8+ T cells with MHC class I/II restriction specificity.

Following positive selection, T cells undergo further screening in the thymic medulla based on the affinity of their TCRs for self‐peptide–MHC complexes [94]. This process, termed negative selection, is mediated by thymic antigen‐presenting cells (APCs), including specialized medullary thymic epithelial cells (mTECs), DCs, and B cells (Figure 3).

T cells exhibiting high‐affinity binding to self‐peptide–MHC complexes undergo developmental arrest and clonal deletion, a process critical for eliminating autoreactive precursors [95]. Thymocytes engaging self‐peptide–MHC complexes with intermediate affinity differentiate into Tregs, which suppress effector T‐cell (Teff) activation [96]. whereas low‐affinity interactions permit positive selection of conventional T cells, which subsequently egress from the thymus into peripheral circulation. Since central tolerance is established exclusively within the thymus, robust negative selection necessitates broad self‐peptide presentation by thymic APCs [97]. mTECs express the autoimmune regulator (Aire) transcription factor, which plays a crucial role in central tolerance by regulating the expression of tissue‐specific antigens [98].

##### 3.2.1.2 B‐Cell Tolerance

In the bone marrow, progenitor cells committed to the B‐cell lineage undergo V(D)J recombination of immunoglobulin germline genes, a process that generates diverse B‐cell antigen receptors (BCRs) essential for adaptive immunity [99]. Despite this diversity serving as the foundation for immune recognition, a substantial proportion (55–75%) of immature B cells exhibit self‐reactivity during development [100]. To establish central tolerance, immature B cells undergo stringent selection through three interconnected mechanisms: clonal deletion, anergy, and receptor editing.

Immature B cells expressing autoreactive BCRs that engage high‐affinity self‐antigens undergo developmental arrest and clonal deletion via apoptosis [101]. In contrast, low‐affinity BCR‐self‐antigen interactions fail to reach the activation threshold required for positive selection, inducing anergy instead [102]. Secondary immunoglobulin gene rearrangement, another tolerance mechanism, modifies autoreactive BCRs to generate variants with reduced self‐antigen affinity [103]. The remaining immature B cells, which do not engage self‐antigens at levels sufficient to trigger central tolerance, are permitted to mature and migrate to peripheral tissues such as the spleen and lymph nodes. In the periphery, these cells rapidly undergo additional tolerance checks to prevent tissue damage [104].

Although central tolerance mechanisms effectively eliminate the majority of self‐reactive T and B cells during thymic and bone marrow development, a subset of autoreactive clones specific for developmentally restricted antigens or inducible antigens escapes central tolerance checks [105]. This residual autoreactivity necessitates a second layer of control: peripheral tolerance, which actively suppresses the activation and effector functions of circulating self‐reactive lymphocytes.

#### 3.2.2 Peripheral Tolerance

Despite the dominant role of the thymus and bone marrow in immune tolerance, some autoreactive lymphocytes escape central tolerance and persist in the periphery, where they may trigger unexpected immune responses. This highlights the critical role of peripheral tolerance mechanisms in preventing autoimmune diseases. Peripheral tolerance, including anergy, deletion, Treg‐mediated suppression, and inhibitory checkpoint molecules (e.g., Cytotoxic T lymphocyte associate protein‐4 [CTLA‐4], Programmed cell death protein‐1 [PD‐1), acts synergistically with central tolerance to ensure that the immune system does not cause tissue damage (Figure 3) [106].

##### 3.2.2.1 Anergy

The fate of T cells is determined by three key signals: (1) TCR engagement with MHC–peptide complexes, (2) costimulatory or coinhibitory receptor interactions, and (3) cytokine receptor signaling. When T cells engage with TCRs without adequate costimulation, they enter a hyporesponsive state termed anergy, characterized by failed proliferation and IL‐2 production [107]. Pre‐exposure to cytokines such as IL‐2 prior to activation and costimulation can also induce anergy. Sckisel et al. [108] demonstrated that preconditioning of T cells by cytokines promotes antigen‐specific tolerance, a strategy with therapeutic potential in transplantation.

B‐cell anergy shares similarities with T‐cell anergy. It arises when B cells engage with BCRs without concomitant T‐cell help (e.g., CD40L interaction) or Toll‐like receptor costimulation, leading to reduced surface immunoglobulin M (IgM)–BCR expression and impaired antibody secretion [109]. Both T‐ and B‐cell anergy lead to peripheral tolerance by limiting autoreactive lymphocyte activation and shortening their lifespan in the absence of costimulatory signals, thereby preventing autoimmune pathology.

##### 3.2.2.2 Peripheral Deletion

The maintenance of peripheral tolerance is dependent on antigen stimulation thresholds, which dictate the survival or functional silencing of self‐reactive lymphocytes. High‐affinity antigen exposure predominantly induces clonal anergy in T and B cells, whereas suboptimal antigen levels trigger apoptotic deletion, effectively purging autoreactive clones from the periphery [110].

The clearance of self‐reactive T cells involves multiple mechanisms. Fas receptor engagement by the Fas–Fas ligand (FasL) and the Bim‐dependent mitochondrial pathway play key roles in the deletion of these cells [111, 112]. At the termination of immune responses, Fas‐mediated apoptosis of peripheral T cells prevents excessive immune response‐induced tissue damage, a process regulated by FasL expressed on activated T cells [113]. This mechanism, known as activation‐induced cell death (AICD), promotes the formation of the death‐inducing signaling complex and activates apoptotic caspase cascades. During T‐cell deletion, upregulated Bim neutralizes the function of antiapoptotic proteins such as Bcl‐2 and cooperates with proapoptotic proteins Bax and Bak to destabilize mitochondrial integrity, thereby maintaining self‐tolerance [114]. In mouse models, mice deficient in Bim or Fas both exhibit impaired T‐cell deletion and defective peripheral tolerance [115, 116, 117]. Similarly, AICD of B cells can also be induced by FasL expressed on T cells, a process dependent on CD40–CD40L engagement to prime B cells for Fas sensitivity [118]. This mechanism is critical for eliminating autoreactive B cells and maintaining immune homeostasis.

##### 3.2.2.3 Regulatory Cells

Diverse regulatory cell populations, including FoxP3^+^ Tregs, FoxP3^–^ Tregs, regulatory B cells, CD8+ Tregs, and γδ Tregs, collaborate to enforce peripheral tolerance and maintain tissue homeostasis [119, 120, 121, 122]. Among these, Treg‐mediated immune tolerance is central to preventing autoimmunity. Tregs, a suppressive CD4+ T‐cell subset, are characterized by high FoxP3 expression and constitutive expression of IL‐2Rα (CD25) and CTLA‐4. Mutations in *FoxP3*, *IL2RA*, or *CTLA4* impair Treg function and lead to systemic autoimmunity [123, 124]. Tregs are categorized into thymus‐derived Tregs (tTregs) and peripherally induced Tregs (pTregs). tTregs undergo selection in the thymus to recognize self‐antigens, whereas pTregs differentiate from naïve CD4+ T‐cells in peripheral tissues on exposure to dietary or microbial antigens in transforming growth factor‐β (TGF‐β)‐rich environments [125]. In the steady state, tTregs remain quiescent while circulating through blood and lymphoid tissues. However, on encountering an antigenic challenge such as tissue inflammation, they rapidly activate, upregulate chemokine receptors (such as CCR4 and CCR5), and migrate to inflamed tissues to suppress autoimmune pathology [126]. Activated CD4+ T‐cells differentiate into pTregs. This transdifferentiation occurs predominantly at mucosal surfaces interfacing with external stimuli [127]. Helios and Neuropilin‐1 are preferentially expressed on tTregs [128, 129]. Overall, pTregs and tTregs work in concert to prevent autoimmunity by suppressing proinflammatory T‐cell‐mediated immune responses.

Tregs are involved in preventing multiorgan autoimmunity through multiple mechanisms, and suppress immune responses to maintain immune homeostasis through diverse pathways, including (1) cell‐contact‐dependent inhibition, (2) cytokine‐mediated suppression, and (3) resource competition.

Direct suppression of Teffs is achieved through multiple contact‐dependent pathways. Tregs constitutively express high levels of CD25 (IL‐2Rα), which acts as an “IL‐2 sink” to deprive neighboring Teffs of this cytokine, which is essential for their survival, leading to Teff apoptosis [130]. Simultaneously, the ectoenzyme pair CD39/CD73 on Treg surfaces degrades extracellular adenosine triphosphate into adenosine, which engages A2a receptors on Teffs to inhibit their proliferation and cytokine production [131].

Modulation of APCs is another mechanism whereby Tregs mediate tolerance. The inhibitory receptor CTLA‐4, which is highly expressed on Tregs, competitively binds B7 molecules (CD80/CD86) on DCs, outcompeting CD28‐mediated costimulation [132]. CTLA‐4 induces DCs to internalize and degrade B7 molecules through trans‐endocytosis, while also activating the immunosuppressive enzyme indoleamine 2,3‐dioxygenase, which leads to local tryptophan depletion [133]. These actions collectively render APCs incapable of fully activating autoreactive T cells, even when they present self‐peptides.

IL‐10, TGF‐β, and IL‐35 form the core cytokine network through which Tregs mediate immune suppression. IL‐10 exerts its immunosuppressive effects by directly inhibiting the activation and proliferation of Teffs and impairing the antigen‐presenting function of APCs. This occurs via activation of the STAT3 signaling pathway, which suppresses inflammatory gene expression and promotes Treg development [134]. IL‐10 drives the conversion of Th1 cells into FoxP3− Tregs [135], and simultaneously inhibits chemokine production, which is essential for Teff migration [136]. TGF‐β is a key regulator of immune tolerance, and TGF‐β deficiency is associated with severe multiorgan inflammation [137]. TGF‐β maintains immune balance by suppressing key transcription factors and cytokine receptors required for Th1 and Th2 differentiation, and promoting Th17 and Th9 development in certain inflammatory contexts [138]. IL‐35, the most recently characterized member of the cytokine network, amplifies immunosuppression through multiple mechanisms, including induction of IL‐10‐producing B10‐regulatory B cells, expansion of IL‐35+ Treg populations, and direct suppression of Th17 cell differentiation via STAT1/STAT3 signaling pathways [139]. These cytokines operate in concert to ensure robust peripheral immune tolerance.

### 3.3 Triggers of Autoimmune Responses

Autoimmune diseases arise owing to a combination of genetic susceptibility, environmental exposures, and breakdowns in immune tolerance.

Genetic predisposition serves as a foundational risk: Polymorphisms in human leukocyte antigen (HLA)/MHC genes (such as HLA‐DR4 in RA) alter self‐antigen presentation, increasing the likelihood of autoreactive T‐cell activation [140, 141]. Non‐HLA genes, such as PTPN22 (affecting lymphocyte signaling) and STAT4 (which modulates IFN responses), further disrupt immune checkpoint regulation [142, 143]. These genetic variants collectively lower the threshold for immune dysregulation but rarely trigger autoimmunity on their own.

Environmental cofactors, including pathogens such as Epstein–Barr virus (EBV) and *Streptococcus pyogenes*, promote autoimmunity by molecular mimicry (e.g., EBV nuclear antigen‐1 cross‐reacts with lupus‐associated autoantigens) or bystander activation during tissue damage [144, 145]. Smoking is also a risk factor for autoimmune disease [146, 147]. Chemical triggers, including silica dust, mercury, and drugs such as procainamide, induce oxidative stress, modify self‐proteins into neoantigens, or dysregulate epigenetic controls on genes responsible for the immune response [148, 149]. Ultraviolet radiation, hormonal fluctuations (e.g., estrogen‐driven lupus flares), and chronic stress exacerbate these effects by causing cytokine imbalances (e.g., elevating IL‐6 and TNF‐α) and impairing Treg suppressive capacity [150, 151, 152].

The interplay of genetic and environmental factors disrupts immune homeostasis. Tregs, essential for restraining autoreactive lymphocytes, become numerically or functionally deficient, whereas DCs and B cells transition toward proinflammatory states. This shift creates a permissive microenvironment in which self‐tolerance erodes, setting the stage for persistent autoreactivity [153]. These systemic imbalances mark the transition from preclinical susceptibility to active autoimmunity, priming the immune system for the uncontrolled inflammation and tissue targeting detailed in the disease progression pathways.

### 3.4 Pathogenic Progression in Autoimmunity

**FIGURE 4:**
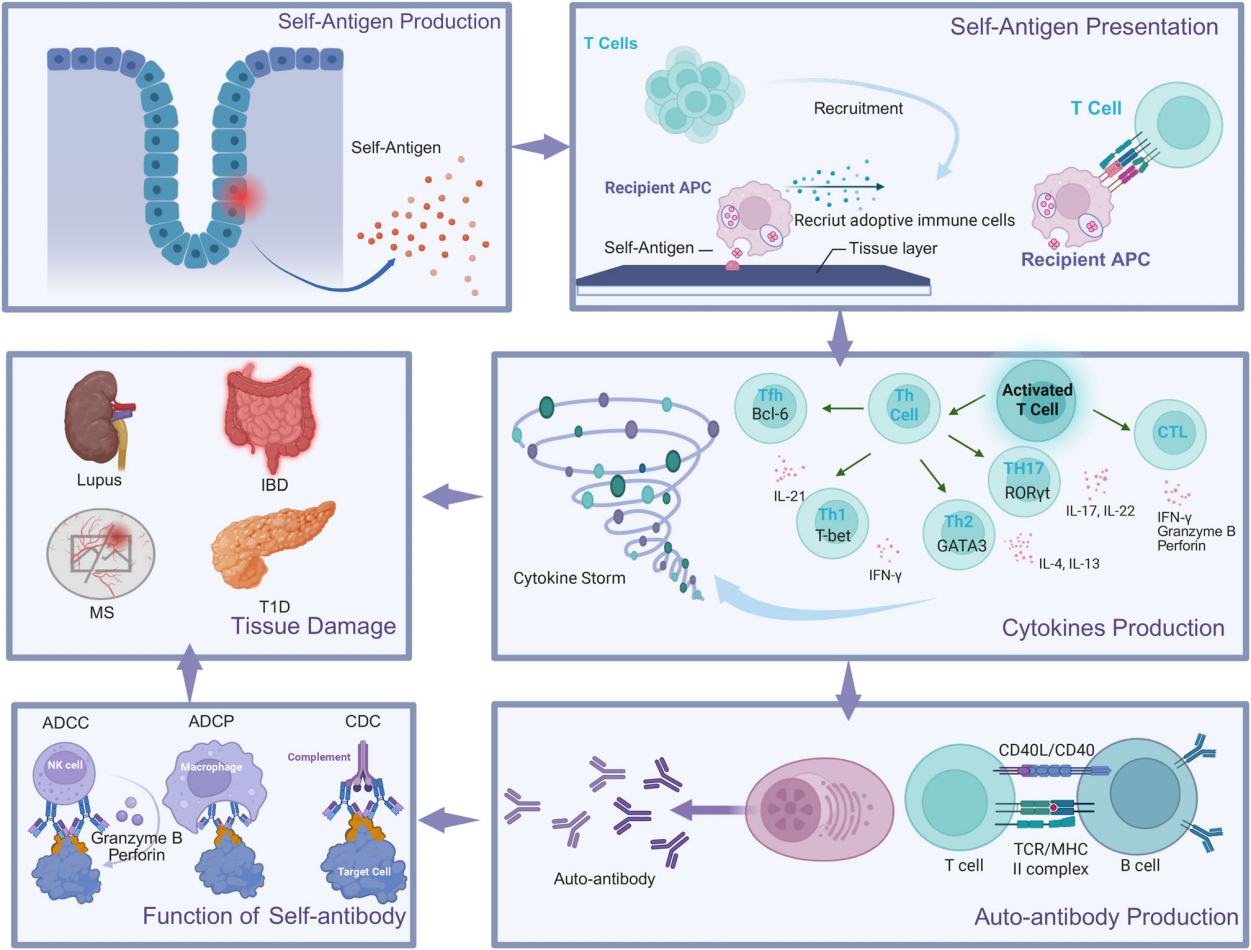
The development of autoimmunity. Under pathogenic conditions, healthy tissues expose self‐antigens to antigen‐presenting cells (APCs), leading to their activation and subsequent recruitment of T cells to specific sites. Following T‐cell migration, APCs capture, process, and present self‐antigens to T cells. On antigen recognition, T cells initiate the expression of key transcription factors induced by various cytokines, driving their differentiation into distinct subsets, including CD4+ T‐helper 1 (Th1) cells, Th2 cells, Th17 cells, T‐follicular helper (Tfh) cells, and CD8+ cytotoxic T lymphocytes (CTLs). These differentiated T cells secrete a broad array of cytokines, generating a cytokine storm that exacerbates tissue damage through a positive feedback loop. Autoantibody production is mediated by the interaction between CD4+ T‐helper cells and B cells. Once autoantibodies bind to target tissues, they induce multiorgan failure via multiple effector mechanisms, including antibody‐dependent cell‐mediated cytotoxicity (ADCC), antibody‐dependent cellular phagocytosis, and complement‐dependent cytotoxicity.

The progression of autoimmune diseases involves a cascade of pathogenic events that extend beyond the initial breakdown of immune tolerance. Although genetic susceptibility and environmental factors prime the immune system for autoreactivity, the ultimate clinical manifestations arise from three interrelated processes: (1) the production of pathogenic autoantibodies, (2) dysregulated cytokine networks, and (3) irreversible tissue injury. These effector phases often coexist and amplify each other, creating self‐perpetuating cycles of inflammation and damage that characterize chronic autoimmunity (Figure 4). The specific pathological outcomes in a given disease context depend on which molecular and cellular pathways dominate, leading to diverse clinical presentations despite shared underlying pathogenic mechanisms.

#### 3.4.1 Autoantibody Production

Autoantibodies play a dual role in physiology and pathology. Under normal conditions, polyreactive autoantibodies produced by B cells aid in clearing cellular debris and microbial components [154]. However, in autoimmune diseases, loss of immune tolerance leads to the generation of high‐affinity pathogenic autoantibodies (e.g., anti‐double‐stranded DNA [anti‐dsDNA] in SLE and anticitrullinated protein antibodies [ACPAs] in RA) through somatic hypermutation and class‐switch recombination [155]. The presence of autoantibodies alone does not necessarily predict disease onset. The pathogenicity of autoantibodies depends on coexisting inflammatory signals. For example, in T1D, only a subset of children with asymptomatic immunoglobulin G (IgG) autoantibodies progress to clinical disease [156].

#### 3.4.2 Cytokine Storm

##### 3.4.2.1 Innate Immune System Activation

The cytokine storm characteristic of many autoimmune diseases often begins with dysregulated activation of innate immune cells. Pattern‐recognition receptors on macrophages and DCs respond to damage‐associated molecular patterns released during cell stress or death, leading to excessive production of proinflammatory cytokines including IL‐6, TNF‐α, and type I IFNs [157]. For example, In SLE, neutrophil extracellular traps containing self‐DNA and antimicrobial proteins provide potent stimuli for sustained IFN production and autoantibody development [158].

##### 3.4.2.2 Adaptive Immune System Amplification

The adaptive immune system amplifies the cytokine cascade through specialized T‐cell subsets. Th17 cells, differentiated under the influence of IL‐6 and TGF‐β, produce IL‐17, which promotes neutrophil recruitment and activation [159]. Follicular helper T (Tfh) cells, through the production of IL‐21, provide critical support for autoreactive B cells in germinal centers, facilitating the production of high‐affinity pathogenic autoantibodies [160]. This coordinated interaction between innate and adaptive immunity creates a self‐sustaining inflammatory environment that drives disease progression.

##### 3.4.2.3 Key Cytokine Pathways

IL‐6 exemplifies the pleiotropic effects of cytokines in autoimmunity. Through activation of the JAK–STAT pathway, IL‐6 promotes Th17 differentiation, B‐cell antibody production, and hepatic acute‐phase responses [161]. In RA, synovial fibroblasts are both sources of IL‐6 and responders to IL‐6, creating an autocrine loop that maintains joint inflammation [162]. Similarly, type I IFNs play a central role in SLE by upregulating hundreds of IFN‐stimulated genes that amplify immune activation and autoantibody production [163].

#### 3.4.3 Tissue Damage

Tissue injury in autoimmunity occurs through three principal mechanisms that frequently coexist and interact. The first mechanism involves immune‐complex deposition in which antibody–antigen aggregates accumulate in tissues such as renal glomeruli or synovial membranes [164]. These complexes activate complement proteins and engage Fc receptors on innate immune cells, triggering inflammatory cascades that recruit neutrophils and monocytes. The subsequent release of reactive oxygen species and proteolytic enzymes directly damages the tissue architecture, and simultaneously increases vascular permeability, which facilitates further leukocyte infiltration [165, 166].

The second mechanism is direct cellular cytotoxicity, which operates through distinct but complementary pathways. Autoreactive CD8+ T cells identify and eliminate target cells through cytotoxic granule exocytosis (perforin/granzyme B) and death receptor interactions (Fas/FasL), a process central to pancreatic β‐cell destruction in T1D [167, 168]. Antibody‐dependent cellular cytotoxicity (ADCC) mediated by NK cells and complement‐mediated lysis is an additional cytotoxic arm, acting in parallel with autoreactive CD8+ cells [169, 170]. These mechanisms collectively contribute to tissue destruction in conditions such as autoimmune cytopenia, in which opsonized blood cells are systematically cleared.

The third mechanism is chronic inflammation, which results in structural remodeling through fibrotic transformation. Sustained cytokine signaling, particularly through TGF‐β and IL‐13, activates resident fibroblasts to deposit excessive extracellular matrix components [171]. This fibrotic response manifests differently in different tissues, for example, as synovial pannus in rheumatoid joints, interstitial fibrosis in AIH, and dermal thickening in SSc. The transition from reversible inflammation to irreversible fibrosis is a critical therapeutic juncture, with TGF‐β regulating this pathological transition [172].

These mechanistic categories are not mutually exclusive but rather form an integrated pathological network. Immune complexes may initiate damage that is amplified by cellular cytotoxicity, whereas both processes fuel the chronic inflammation that drives fibrosis. The relative contribution of each pathway determines the rapidity of progression and pattern of tissue injury, explaining clinical heterogeneity among autoimmune disorders.

## 4 Approaches to Diagnosing Autoimmune Diseases

The diagnosis of autoimmune diseases relies on a multidisciplinary approach, integrating detailed clinical evaluation, laboratory testing, imaging studies, and histopathological confirmation. Given the heterogeneous nature of autoimmune disorders and their frequent multiorgan involvement, a combination of detecting specific biomarkers and exclusionary testing is essential for accurate diagnosis. This section summarizes current diagnostic strategies and their clinical applications.

### 4.1 Clinical History and Physical Examination

A thorough medical history and physical examination are fundamental in the diagnostic workup of autoimmune diseases. Patients often present with nonspecific symptoms such as fatigue, low‐grade fever, and weight loss, but certain disorders exhibit characteristic clinical features as detailed in the previous section. For example, SLE may manifest with malar rash, photosensitivity, arthritis, and renal involvement [11], whereas RA typically presents with symmetric small‐joint swelling and morning stiffness [8]. Neurological symptoms, such as vision loss or motor weakness, may suggest demyelinating lesions due to MS [57]. Additionally, family history, history of prior infections, and medication use (e.g., drug‐induced lupus from antiarrhythmic drugs) should be carefully assessed to aid the differential diagnosis.

### 4.2 Laboratory Investigations

Laboratory testing, including testing levels of nonspecific inflammatory markers and disease‐specific autoantibodies, plays a key role in the diagnosis of autoimmune diseases.

#### 4.2.1 Nonspecific Indicators

##### 4.2.1.1 Biochemical Markers

Biochemical alterations directly reflect organ‐specific dysfunction in autoimmune diseases. For example, elevated hepatic transaminases (alanine transaminase and aspartate transaminase) indicate hepatocellular injury in AIH [173], whereas persistent hyperglycemia signals pancreatic β‐cell destruction in T1D. Hypocomplementemia (reduced C3/C4 levels) is a diagnostic hallmark of active SLE nephritis, correlating with renal immune complex deposition and disease activity [174].

##### 4.2.1.2 Inflammatory Markers

Acute‐phase reactants provide nonspecific measures of systemic inflammation. C‐reactive protein and erythrocyte sedimentation rate are routinely used to monitor inflammatory burden [175]. However, their diagnostic utility is limited owing to their lack of disease specificity, as elevations may occur in infections, malignancies, and other inflammatory conditions.

#### 4.2.2 Specific Autoantibodies

Testing for autoantibodies plays a key role in the diagnosis of autoimmune diseases, with different autoantibodies associated with different diseases.

##### 4.2.2.1 Systemic Lupus Erythematosus

Antinuclear antibodies (ANA) are pivotal for the diagnosis of SLE, exhibiting high sensitivity (90–95%), though they are not exclusive to SLE. Anti‐dsDNA antibodies, with a specificity of 96%, serve as supportive evidence for SLE diagnosis and are linked to disease activity, particularly in lupus nephritis. The presence of SLE‐specific anti‐Smith antibodies, despite their low sensitivity (5–30%), is highly specific (99%) and confirms the diagnosis. Additionally, antinucleosome antibodies are associated with renal involvement, making their detection valuable for early identification of lupus nephritis, with a specificity of 94% and a positive likelihood ratio of 13.81. These antibodies collectively enhance diagnostic accuracy and help monitor disease progression in patients with SLE [174, 176].

##### 4.2.2.2 Rheumatoid Arthritis

Serological diagnosis of RA relies on two principal markers: rheumatoid factor (RF) and ACPAs (e.g., anticyclic citrullinated peptide [CCP]). ACPA exhibits higher specificity than RF, particularly in early disease stages. Recently, antibodies targeting modified proteins, such as carbamylated or acetylated antigens (e.g., anticarbamylated protein antibodies), have shown diagnostic potential, though further validation of their clinical utility is needed [177, 178].

##### 4.2.2.3 Type 1 Diabetes

The autoimmune origin of T1D is supported by the presence of islet autoantibodies, which serve as key markers for the disease. These autoantibodies target specific pancreatic islet‐associated antigens, such as glutamic acid decarboxylase (GAD65), islet antigen‐2 (IA‐2), and zinc transporter‐8 (ZnT8). In children, the detection of these autoantibodies demonstrates high diagnostic sensitivity for T1D, with studies indicating that their combined presence significantly enhances the accuracy of disease prediction and diagnosis. Notably, the predictive power of these autoantibodies extends beyond mere detection, as their characteristics—including epitope specificity, affinity, and longitudinal patterns—further refine risk stratification and progression assessment in preclinical stages of T1D [179, 180].

##### 4.2.2.4 Inflammatory Bowel Disease

In IBD, measuring autoantibodies is useful for determining the subtype. Antiglycoprotein 2 antibodies are more prevalent in stricturing Crohn's disease, whereas perinuclear antineutrophil cytoplasmic antibodies (p‐ANCA) are strongly associated with ulcerative colitis. Measurement of both antiglycoprotein 2 antibodies and p‐ANCA enhances diagnostic accuracy [181].

##### 4.2.2.5 Hashimoto's Thyroiditis

Antithyroid peroxidase (anti‐TPO) and antithyroglobulin (anti‐Tg) antibodies are almost always elevated in HT. Anti‐TPO antibody levels are often elevated before the onset of thyroid dysfunction [182].

##### 4.2.2.6 Autoimmune Hepatitis

Subtyping of AIH is guided by distinct autoantibody profiles: type 1 is characterized by antinuclear (ANA) and antismooth muscle antibodies, whereas type 2 is defined by anti‐liver–kidney microsomal type 1 (anti‐LKM1) antibodies. These markers are also useful for monitoring response to treatment and predicting prognosis [183].

### 4.3 Imaging Techniques

In autoimmune diseases, imaging is indispensable for assessing organ damage, disease extent, and treatment response. Imaging modalities range from conventional radiography to advanced functional imaging, each offering unique insights into pathophysiology and guiding precision medicine. Below, we highlight key applications across representative autoimmune disorders.

#### 4.3.1 Rheumatoid Arthritis

Radiography remains key to the detection of structural damage, including joint space narrowing and bone erosion, which correlate with disease severity and progression. However, its limited sensitivity for early inflammation has led to the use of ultrasound and magnetic resonance imaging (MRI). High‐resolution ultrasound enables dynamic visualization of synovial hypertrophy, and power Doppler signals (which indicate vascularization), enabling real‐time assessment of inflammatory activity. MRI further enhances detection of bone marrow edema and pre‐erosive changes, facilitating early intervention before irreversible joint damage occurs. These tools are critical for implementing targeted treatment strategies and monitoring responses to biologic therapies [177, 178].

#### 4.3.2 Multiple Sclerosis

Brain MRI is key for diagnosing MS, revealing periventricular white matter lesions (Dawson's fingers) and cortical demyelination. Gadolinium‐enhanced T1‐weighted sequences distinguish active plaques (indicating blood‐brain barrier disruption) from chronic lesions, enabling the identification of RR MS. Advanced techniques such as diffusion tensor imaging and magnetization transfer ratio quantify axonal loss and myelin integrity, enabling the prediction of prognosis. MRI also guides therapeutic decisions, such as escalating from immunomodulators (e.g., IFN‐β) to B‐cell‐depleting agents (e.g., ocrelizumab) in aggressive cases [58].

#### 4.3.3 Inflammatory Bowel Disease

MRI and CT enterography have become important noninvasive alternatives to invasive endoscopy for differentiating Crohn's disease from ulcerative colitis, especially in certain clinical situations such as when patients are unable or unwilling to undergo invasive procedures. MRI is better than CT at detecting soft‐tissue contrast, enabling the differentiation between transmural inflammation (a hallmark of Crohn's disease) and mucosal‐limited involvement (ulcerative colitis), while avoiding exposing the patient to ionizing radiation. Key findings include bowel wall thickening, ulceration, and strictures. Fat wrapping on MRI predicts fibrostenotic complications. CT is reserved for acute settings (e.g., intestinal perforation). Both modalities are useful for monitoring response to treatment (e.g., vedolizumab‐induced mucosal healing) and for surgical planning [181].

#### 4.3.4 Hashimoto's Thyroiditis

Thyroid ultrasound reveals diffuse hypoechogenicity (due to lymphocytic infiltration) and pseudonodules, often preceding elevations in serologic markers such as anti‐TPO antibodies. Doppler imaging distinguishes hypothyroid (reduced vascularity) in the hashitoxicosis phases (transient hypervascularity). Ultrasound can be used to screen for malignant transformation (e.g., papillary carcinoma) and can be used to guide fine‐needle aspiration in suspicious cases [182].

#### 4.3.5 Autoimmune Hepatitis

Although liver biopsy remains the gold standard for diagnosis, ultrasound and MRI are useful for noninvasive monitoring. Ultrasound detects hepatomegaly and coarse echotexture in early stages, whereas MRI identifies regenerative nodules and fibrosis patterns (e.g., the “honeycomb” sign). Transient elastography (FibroScan) quantifies stiffness, enabling biopsy frequency to be reduced. In advanced AIH, MRI cholangiography excludes overlap with PSC [183].

### 4.4 Histopathological Evaluation

Tissue biopsy remains the gold standard for diagnosing many autoimmune conditions. In SLE, skin biopsy may show immunoglobulin deposition at the dermoepidermal junction (lupus band test), whereas in AIH, liver biopsy typically reveals interface hepatitis and plasma cell infiltration. Colon biopsy distinguishes Crohn's disease (noncaseating granulomas) from ulcerative colitis (continuous mucosal inflammation). Renal biopsy is indispensable for classifying lupus nephritis and guiding treatment.

Most autoimmune diseases are diagnosed using internationally accepted criteria, such as the 2019 European League Against Rheumatism/American College of Rheumatology (EULAR/ACR) classification for SLE, and the 2010 ACR/EULAR criteria for RA. These frameworks combine clinical, serological, and imaging findings to enhance diagnostic accuracy. However, some conditions (e.g., undifferentiated connective tissue disease) may not fulfill the criteria, necessitating longitudinal follow‐up to make the diagnosis. Furthermore, diagnostic challenges persist, including false‐positive/false‐negative antibody results and the absence of specific biomarkers for certain diseases (e.g., seronegative spondyloarthropathies). Emerging technologies such as multiomics (proteomics, metabolomics) and artificial intelligence hold promise for refining diagnostic precision.

## 5 Current Therapeutic Landscape in Autoimmune Diseases

**FIGURE 5:**
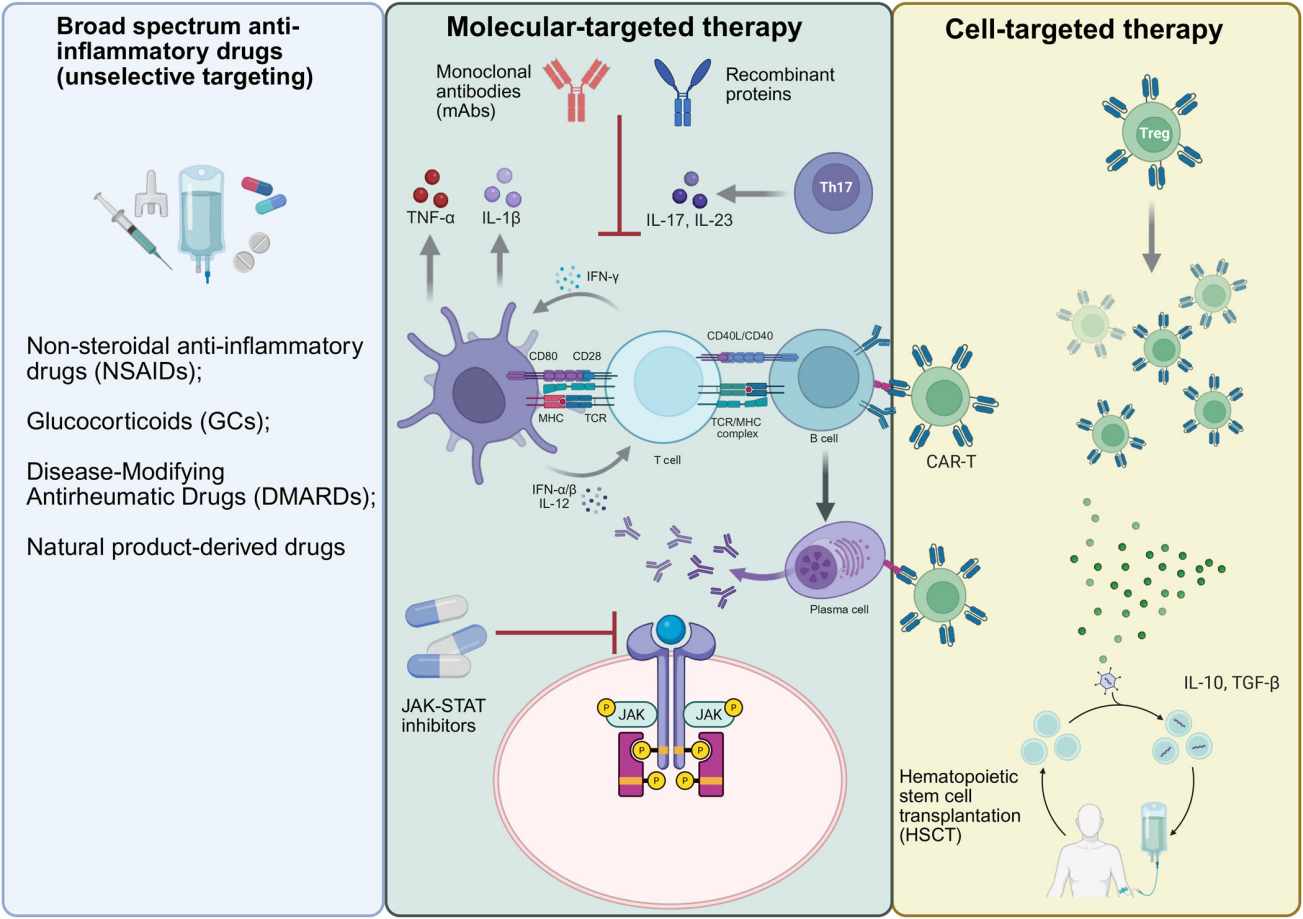
Therapeutic strategies for autoimmune diseases. Current treatments include broad‐spectrum anti‐inflammatory drugs (such as nonsteroidal anti‐inflammatory drugs and glucocorticoids), molecular‐targeted therapies (such as JAK inhibitors and anti‐TNF biologics), and emerging cell‐targeted approaches (such as CAR‐T therapy and Treg transfer). These strategies range from nonspecific immunosuppression to precise immune modulation, with newer therapies aiming to restore immune tolerance while minimizing side effects. The figure illustrates key drug classes, their molecular targets, and clinical applications across different autoimmune disorders.

The onset of autoimmune diseases is caused mainly by the abnormal recognition and response of the immune system to self‐antigens, usually accompanied by strong inflammatory and immune responses. Monotherapy for autoimmune diseases can be divided into three types: broad‐spectrum anti‐inflammatory drugs, molecular‐targeted therapy, and cell‐targeted therapy. Broad‐spectrum anti‐inflammatory drugs alleviate autoimmune reactions by inhibiting the activity of various cells and molecules in the immune system, thereby achieving therapeutic effects. Molecular‐targeted drugs are a class of drugs that act on specific molecules or signaling pathways, with the aim of precisely intervening in the occurrence and development of diseases and reducing adverse effects on normal cells. Molecular‐targeted therapy is divided into small‐molecule‐targeted drugs and large‐molecule‐targeted drugs. Small‐molecule targeted drugs, such as JAK inhibitors, exert anti‐inflammatory effects by blocking specific signaling pathways [184]. Large‐molecule‐targeted drugs, such as monoclonal antibodies (mAbs) (such as anti‐CD20 antibody [185] and anti‐TNF‐α antibody [186]), regulate immune responses by specifically neutralizing target molecules or cell depletion strategies. Cell‐targeted therapy achieves therapeutic goals by specifically identifying and eliminating abnormal immune cells and has shown great potential in the treatment of certain refractory autoimmune diseases, making it an emerging therapeutic approach (Figure 5).

### 5.1 Broad‐Spectrum Anti‐Inflammatory Drugs (Unselective Targeting)

Nonsteroidal anti‐inflammatory drugs (NSAIDs) are commonly used in the initial treatment of autoimmune diseases because of their rapid anti‐inflammatory and analgesic effects. They function by inhibiting cyclooxygenase (COX), which has two isoforms: COX‐1 and COX‐2 [187, 188]. COX‐1 is constitutively expressed in most tissues and is responsible for producing prostaglandins that maintain normal physiological functions, such as protecting the gastrointestinal mucosa and regulating renal blood flow [189]. In contrast, COX‐2 is inducible and primarily involved in prostaglandin synthesis during inflammation [190]. However, NSAIDs only alleviate symptoms and do not address the underlying cause of the disease or control its activity and progression. Their nonspecific inhibition of the COX pathway affects prostaglandin production throughout the body and lacks cellular or molecular specificity. Traditional NSAIDs, such as ibuprofen and naproxen, inhibit both COX‐1 and COX‐2, effectively reducing inflammation but increasing the risk of gastrointestinal and renal adverse reactions [191]. In comparison, selective COX‐2 inhibitors such as celecoxib have less gastrointestinal toxicity. For example, in a randomized, controlled comparative study, the incidence of ulcers was 2.6% among patients taking celecoxib [192]. Celecoxib is widely used for treating RA and ankylosing spondylitis (AS), effectively relieving pain and improving patients' quality of life. However, the elevated risk of cardiovascular disease, and the withdrawal of rofecoxib owing to the increased risk of thrombotic events, suggests that COX‐2 inhibitors should be used with caution [193].

Glucocorticoids are essential for treating severe autoimmune disease and refractory cases, offering broad anti‐inflammatory and immune‐regulatory effects. They control acute lesions by reducing key proinflammatory cytokines such as IL‐1 and IL‐6, inhibiting IL‐2 production and proliferation of T cells, inhibiting neutrophil and monocyte chemotaxis, and lessening lysosomal enzyme release [194, 195, 196]. Extensive studies have confirmed their effectiveness for relieving symptoms, enhancing physical function, and improving quality of life [197]. However, glucocorticoids have serious adverse effects, including post‐withdrawal relapses, inability to prevent bone destruction, and increased risk of infection, cardiovascular and cerebrovascular disease, and osteoporosis [198]. Currently, glucocorticoids remain fundamental for inducing remission in RA and SLE [199, 200]. In chronic management, glucocorticoids are often combined with disease‐modifying antirheumatic drugs (DMARDs) to rapidly control disease activity while waiting for the DMARDs to take effect [201].

First‐line synthetic DMARDs include methotrexate (MTX), which is used for treating RA, leflunomide (LEF), and azathioprine [202]. Cyclophosphamide and mycophenolate mofetil are reserved for treating severe autoimmune diseases (such as lupus nephritis) [203]. The chemical structure and pharmacological mechanism of action of traditional DMARDs differ. MTX inhibits dihydrofolate reductase and reduces tetrahydrofolate formation, blocking DNA synthesis, and can delay joint damage and enable repair of bone damage, so it is suitable for use in treating active RA [204]. LEF mainly inhibits dihydroorotate dehydrogenase activity and affects lymphocyte pyrimidine synthesis, which can improve symptoms in patients with RA [205]. However, both MTX and LEF have slow onset of action, leading to gradual reduction in the symptoms and signs after several weeks or months, and thus require long‐term continuous administration.

Natural product‐derived drugs can have both broad‐spectrum anti‐inflammatory properties and certain molecular‐targeted effects. *Tripterygium wilfordii*, a traditional Chinese medicinal herb, contains active compounds such as celastrol, which exhibit potent anti‐inflammatory effects [206]. Celastrol inhibits the NLRP3 inflammasome, a key component in triggering the inflammatory response [207]. Preclinical studies have demonstrated that *Tripterygium wilfordii* can significantly reduce synovitis in RA models, highlighting the value of traditional medicine in treating inflammation [208]. Cp‐25, a novel derivative of paeoniflorin, is under investigation as an immunomodulator. It has been found to regulate the B‐cell‐activating factor (BAFF)‐TNF‐α pathway and to target TNF receptor‐associated factor 2 (TRAF2) and p52 in the NF‐κB signaling axis without causing B‐cell exhaustion. This selective effect preserves normal B‐cell function while inhibiting the pathological immune response [209].

### 5.2 Molecular‐Targeted Therapy

The rise of molecular‐targeted therapy has transformed the treatment of autoimmune disease treatment from broad‐spectrum inhibition to precise intervention. This kind of therapy can effectively control inflammation and significantly reduce systemic toxicity by specifically targeting key molecules in pathogenic signaling pathways, providing patients with better treatment options.

The emergence of targeted small‐molecule inhibitors has changed the therapeutic landscape of autoimmune diseases and provided a new mechanism of action for oral treatment. JAK–STAT inhibitors are a major advance in this category. The JAK–STAT pathway is essential for the signaling of many cytokines and growth factors involved in immune regulation. Tofacitinib, a selective inhibitor of JAK1 and JAK3, is effective in RA treatment [210]. In the RA beam trial, baricitinib, which inhibits JAK1 and JAK2, showed an advantage over adalimumab in patients with MTX‐resistant RA [184]. Phosphodiesterase 4 (PDE4) inhibitors such as apremilast provide another targeting method by increasing intracellular cyclic adenosine monophosphate (cAMP) levels, thereby regulating the IL‐23/th17 axis [211]. This mechanism is particularly relevant in psoriasis and psoriatic arthritis, in which Th17‐mediated inflammation plays a central role. Clinical trials showed that 33% of patients with psoriasis improved the psoriasis area and severity index (PASI75) by 75% after 16 weeks of apremilast treatment [212].

By targeting key molecules with high affinity, mAbs show unique value in treating severe or refractory autoimmune disease. They can accurately recognize and bind specific cytokines, receptors, or cell surface molecules, thus achieving precise regulation of the immune response process. TNF‐α antagonists represented by adalimumab, infliximab, and golimumab effectively inhibit proinflammatory signaling by blocking the interaction between TNF‐α and its receptors, significantly reducing joint destruction and skin inflammation in diseases such as RA, psoriatic arthritis, and IBD [186, 213]. Rituximab decreases B‐cell‐mediated autoimmune responses by targeting CD20 molecules on B cells and is used in the treatment of RA and SLE [185]. Belimumab targets BAFF to reduce cell‐mediated autoimmune responses [214]. Inebilizumab rapidly depletes CD19+ B cells by binding to CD19 antigen, thereby inhibiting the inflammatory response [215]. Secukinumab is suitable for the treatment of psoriasis and psoriatic arthritis in adults who have had an inadequate response to conventional synthetic DMARDs, and for treating axial spondyloarthritis, including AS and nonradiographic axial spondyloarthritis by targeting IL17A [216]. Bimekizumab is the first dual targeting IL‐17A/F antibody, and the 16‐week American College of Rheumatology 50% improvement rate (ACR50) in TNF‐α inhibitor‐refractory psoriatic arthritis was 46% in one study [217]. Tocilizumab and Sarilumab, which target IL‐6 receptors, are approved for treating moderate to severe RA [218]. Ulinumab is a fully human mAb against IL‐12 and IL‐23 receptors, which can block intracellular signal transduction and avoid inflammatory events and T‐cell activation [219]. Guselkumab (Tremfya), a mAb targeting IL‐23, is a fully human dual‐effect mAb. It can block IL‐23 activity and bind to the CD64 receptor on IL‐23‐producing cells. It has been approved for the treatment of moderate to severe plaque psoriasis and active psoriatic arthritis [220]. Mirikizumab (Omvoh), a humanized IgG4 anti‐IL‐23p19 mAb, inhibits the IL‐23 pathway by targeting the p19 subunit of IL‐23 and is used to treat ulcerative colitis [221]. The IL‐1β neutralizing antibody, kanamab, is used for the treatment of autoinflammatory diseases such as systemic juvenile idiopathic arthritis [222]. These mAbs, through precise targeting, enhance therapeutic effects and reduce adverse reactions, offering better options for patients with autoimmune disease.

Recombinant proteins can achieve multitarget immune regulation by modulating the biological activity of endogenous proteins through molecular simulation or functional regulation. Among them, Fc fusion protein‐engineering‐technology significantly prolongs serum half‐life and enhances therapeutic effectiveness through IgG constant region fusion [223]. The currently established recombinant protein therapies include: (1) the IL‐1 receptor antagonist, anakinra, which competitively inhibits the binding of IL‐1α/β to type I receptors and exerts anti‐inflammatory effects in RA and cold pyridine‐related periodic syndrome [224]; (2) the TNF‐α inhibitor, etanercept, which neutralizes the proinflammatory factor TNF‐α in the form of soluble receptors and is widely used in the treatment of autoimmune diseases [225]; (3) the CTLA‐4–Fc fusion protein, abatacept, whose extracellular domain can block the CD28–CD80/86 costimulatory pathway, thereby inhibiting T‐cell activation [226]; (4) the dual inhibitor of the B lymphocyte stimulator (BLyS)‐A PRoliferation‐Inducing Ligand (APRIL), atacicept, which regulates B‐cell differentiation and autoantibody production by targeting key B‐cell activation factors BLyS and APRIL, and has unique value in the treatment of SLE and immunoglobulin A nephropathy [227, 228]. Research on the molecular mechanisms of these treatment strategies has confirmed that recombinant proteins can provide highly targeted biological treatment options for autoimmune diseases by precisely intervening in the activation pathways and cytokine networks of immune cells, including T cells, B cells, and APCs.

### 5.3 Cell‐Targeted Therapy

Cell‐targeted therapy is an emerging field in the treatment of autoimmune diseases, which achieves precise regulation of immune responses by specifically targeting subpopulations of immune cells.

Hematopoietic stem cell transplantation (HSCT) is an advanced method to treat autoimmune diseases by clearing abnormal immune cells and rebuilding the normal immune system. HSCT usually includes three steps: immune elimination, hematopoiesis, and immune reconstitution [229]. Before transplantation, patients received high‐dose chemotherapy or radiotherapy to clear abnormal immune cells, followed by infusion of autologous or allogeneic hematopoietic stem cells, which engraft in the bone marrow and differentiate into new blood cells, including immune cells, thereby rebuilding the immune system. Initial lymphocyte depletion can reduce the immune memory pool, including autoreactive clones, followed by profound immune renewal through hematopoiesis and regeneration of the immune system [230]. This approach offers the possibility of long‐term remission of autoimmune diseases. The stem cell source of HSCT can be patients themselves (autologous transplantation) or a matched donor (allogeneic transplantation) [231]. Autologous transplantation avoids graft‐versus‐host disease (GVHD) but may lead to recurrence due to stem cell defects. For example, in SSc, autologous HSCT can significantly improve skin score and lung function, but some patients still experience fluctuations in their autoantibody spectrum, indicating incomplete establishment of immune tolerance [232, 233]. In MS, autologous HSCT can lead to long‐term remission and is more effective than drugs such as fingolimod and ocrelizumab, especially in patients with high disease activity [234, 235]. Allogeneic transplantation requires long‐term immune suppression and carries a high risk of GVHD. New preconditioning regimens such as antibody–drug conjugates can reduce myelotoxicity, promote donor stem cell implantation, and provide a safer option for treating malignant diseases such as acute leukemia [236]. In addition, HLA‐DQ heterodimers may affect the immune response after transplantation and need to be included in the evaluation of typing [237].

Chimeric antigen receptor (CAR) T‐cell therapy is a clinically validated method of cellular immunotherapy. Through genetic engineering, patients' cells are transformed to express specific CAR molecules, which can target pathogenic cells that clear specific antigens. In recent years, this technology has been extended to the field of autoimmune diseases, especially autologous CAR‐T cell therapy targeting CD19 antigen [214]. For example, in a clinical trial of pediatric SLE, 20 patients treated with CD19–CAR‐T achieved clinical improvement, and the Systemic Lupus Erythematosus Disease Activity Index 2000 (SLEDAI‐2K) score of 15 patients fell below 4 within 3 months [238]. Deep depletion of B cells, including elimination of self‐reactive B‐cell clones, is considered a prerequisite for successful CAR‐T cell therapy targeting CD19 or B‐cell‐maturation antigen (BCMA) [238]. Therefore, only diseases driven by B cells, such as SLE, idiopathic inflammatory myopathies, and SSc respond to CAR‐T cell therapy [239, 240], and CAR‐T cell therapy targeting B cells has little effect on chronic inflammatory diseases lacking pathogenic B cells, such as psoriasis, IBD, and spinal arthritis [241]. In addition, researchers have developed chimeric autoantibody receptor T cells (CAAR‐T) technology to precisely target pathogenic B‐cell subsets and reduce their impact on normal B cells. Preclinical studies have shown that CAAR‐T can significantly reduce the levels of autoantibodies in patients with SLE and also reduce the risk of infection caused by B‐cell depletion [242, 243]. In addition, BCMA‐CD19 bispecific CAR‐T cell therapy showed significant therapeutic effects in patients with SLE/LN. All patients who received sufficient initial dose therapy achieved asymptomatic and drug‐free remission at 46 months of follow‐up [244]. These advances indicate that CAR‐T therapy has broad application prospects in the treatment of autoimmune diseases. Future research will further optimize the design of CAR‐T cells and improve their therapeutic effectiveness and safety.

Tregs play a key role in maintaining immune tolerance and preventing autoimmune responses. Tregs mainly inhibit the activation and proliferation of Teffs by secreting inhibitory cytokines such as IL‐10 and TGF‐β, as well as through direct cell‐cell contact [245, 246]. In recent years, immunotherapy targeting Tregs has shown great potential in the treatment of autoimmune diseases [248], and adoptive‐transfer Treg therapy has shown good therapeutic effects for treating various autoimmune diseases. For example, in a clinical study of T1D, adoptive‐transfer of Tregs significantly prolonged the survival time of pancreatic β‐cells [248, 249]. In addition, in animal models of diseases such as SLE and Crohn's disease, adoptive‐transfer of Treg therapy has been shown to reduce disease manifestations and inflammatory responses [250, 251]. These studies have shown that engineering Treg cells can enhance their function and specificity, thereby improving the accuracy and effectiveness of treatment. Currently, researchers are developing antigen‐specific Treg therapies. This method aims to expand or genetically engineer Tregs in vitro so that they can specifically recognize and respond to self‐antigens, thereby more effectively inhibiting autoimmune responses in vivo [252].

In recent years, an increasing number of studies have revealed that gut microbiota affects immune homeostasis by regulating the differentiation of T and B cells. For example, the amplification of specific gut microbiota in patients with SLE is directly related to the production of autoantibodies, such as anti‐dsDNA antibodies [253, 254, 255]. In experimental autoimmune encephalomyelitis (EAE), overactivation of dopamine D2 receptors in intestinal epithelial cells leads to dysbiosis of the microbiota, which exacerbates central nervous system inflammation through activation of microglia [256, 257]. Short chain fatty acids, such as butyric acid, regulate Treg differentiation and alleviate inflammatory responses in EAE and RA by inhibiting histone deacetylase activity [258, 259]. Probiotics such as oligofructose can selectively promote the proliferation of anti‐inflammatory bacteria and restore Th17/Treg balance [260, 261]. Clinical studies have shown that fecal microbiota transplantation has a regulatory effect on Th1/Th17 response in IBD and psoriasis [262, 263]. Specific antibiotics (such as vancomycin) alleviate the severity of EAE by clearing proinflammatory bacteria (such as *Clostridium*), but long‐term use may exacerbate bacterial dysbiosis [257, 264]. Intestinal microbiota intervention provides a novel therapeutic strategy for autoimmune diseases by regulating immune balance, repairing intestinal barriers, and targeting metabolic pathways. In the future, it may be possible to provide personalized therapies based on the patient's microbiota, but this still requires further research.

In summary, the current monotherapy for autoimmune diseases still focuses on broad‐spectrum anti‐inflammatory and molecular targeting. Cell‐targeted therapy is still in the clinical trial stage, and many potential issues still need to be resolved. A summary of treatment and drug classification is provided in Table 1. The use of combination therapy is increasing [265, 266]. However, the individualized assessment of combination therapy, such as molecular typing of immune cells and biomarker detection, currently remains a major challenge.

**TABLE 1:** Targeted therapy for autoimmune diseases.

Drug category	Target	Immunosuppressive mechanism	Representative drugs	Diseases treated
Broad‐spectrum anti‐inflammatory drugs	Unselective targeting	Unselective COX‐1 and COX‐2 inhibitors	Naproxen	RA, AS [267]
			Ibuprofen	RA [268]
		Selective COX‐2 inhibitors	Celecoxib	RA [269]
		Inhibition of immune cells and inflammatory factors	Glucocorticoids	RA, SLE, CGN [270]
		Blocking of DNA synthesis and interfere cell proliferation	Methotrexate	RA, Ps, PsA [271, 272, 273]
			Leflunomide	RA [271]
			Azathioprine	RA, SLE, AIHA [12, 274, 275]
			Cyclophosphamide	RA, SLE [12, 276]
			Mycophenolate mofetil	RA, SLE, MG [12, 277, 278]
		Inhibition of NLRP3 inflammasome	Tripterygium glycosides	RA, SLE, SLEN [279, 280, 281]
		Regulation of the BAFF–TNF‐α pathway Targeting of TRAF2 and p52 in the NF‐κB pathway	Cp‐25	RA (preclinical) [282]
Molecular‐targeted therapy	IAK1, JAK3	Control of inflammation by inhibiting JAK activity	Tofacitinib	RA, Ps, UC [283]
	JAK1, JAK2		Baricitinib	RA, UC [283]
	PDE4	Regulation of the IL‐23/Th17 axis by increasing intracellular cAMP levels	Apremilast	Ps, PsA [284]
	TNF‐α	Reduction of inflammation by inhibiting TNF‐α	Adalimumab	RA, AS, Ps, PsA, CD [271, 285–287]
			Infliximab	RA, IBD, Ps [271, 286, 288]
			Golimumab	RA, Ps, AS [271, 285, 286]
			Etanercept	RA, AS [271, 286]
	CD20	B lymphocyte depletion by inhibiting CD20 on B lymphocytes	Rituximab	RA [271]
	CD19	CD19+ T‐cell depletion by inhibiting CD20 on B lymphocytes	Inebilizumab	NMOSD [289]
	CD28	Regulation of T‐cell activity	Abatacept	RA, SLE [271]
	BAFF	Inhibition of B‐cell activity by targeting BAFF	Belimumab	SLE [290]
	IL‐17A	Reduced inflammation by inhibiting IL‐17A	Secukinumab	AS, Ps [291, 292]
	IL‐17A/F	Reduced inflammation by inhibiting both IL‐17A and IL‐17F	Bimekizumab	Ps, PsA [292]
	IL‐23	Inhibition of Th17 function by blocking the production and inhibiting the activity of IL‐23	Guselkumab	SLE [293]
			Mirikizumab	UC, IBD [294, 295]
	IL‐1R	Prevention of IL‐1α and IL‐1β binding to IL‐1 receptor	Anakinra	RA [296]
	IL‐1β	Binding to IL‐1β to prevent IL‐1β from binding to IL‐1 receptor	Canakinumab	CAPS [297]
	BLyS and APRIL	Regulation of B‐cell differentiation and autoantibody production by inhibiting both BLyS and APRIL	Atacicept	lgAN, SLE (phase II trials) [227, 228]
Cell‐targeted therapy	Hematopoietic stem cells	Abnormal immune cells are removed and autologous or allogeneic hematopoietic stem cells are injected to redifferentiate immune cells to rebuild the immune system	Hematopoietic stem cell transplantation	MS (phase II trials), IBD (phase III trials), SSc (phase III trials) [298]
	CD19	Use genetic engineering techniques to modify patients’ T cells to target pathogenic cells expressing specific antigens	CAR‐T immunotherapy	SLE (phase I trials), MS (phase I trials) [299, 300]
		Targeting of pathogenic B cells more precisely on the basis of CAR‐T to reduce the effect of therapy on normal B cells	CAAR‐T immunotherapy	SLE (preclinical studies) [242, 243]
	BCMA‐CD19	Targeting of both CD19 and BCMA to inhibit B cells and plasma cells	CAR‐T immunotherapy	SLE (phase I trials) [244]
	Tregs	Engineering of Tregs to enhance their function	Adoptive cell transfer therapy	SLE, IBD (preclinical studies) [250, 251]
		Amplification or genetic engineering of Tregs in vitro so that they can specifically recognize and respond to autoantigens	Antigen‐specific Treg therapy	precilinical studies [252]

*Abbreviations*: AIHA, autoimmune hemolytic anemia; AS, ankylosing spondylitis; CAPS, cryopyrin‐associated periodic syndromes; CD, Crohn's disease; CGN, chronic glomerulonephritis; IBD, inflammatory bowel disease; JIA, juvenile idiopathic arthritis; lgAN, immunoglobulin A nephropathy; MG, myasthenia gravis; NMOSD, neuromyelitis optica spectrum disorder; Ps, psoriasis; PsA, psoriatic arthritis; RA, rheumatoid arthritis; SLE, systemic lupus erythematosus; UC, ulcerative colitis.

## 6 Discussion and Conclusion

The pathogenesis of autoimmune diseases is multifaceted, involving genetic susceptibility, environmental triggers, and breakdown of immune tolerance. Central tolerance mechanisms in the thymus and bone marrow eliminate autoreactive T and B cells, whereas peripheral tolerance relies on Tregs and the indication of anergy. However, defects in these processes—such as impaired Treg function or aberrant cytokine signaling—can lead to autoimmunity. Recent advances in molecular‐targeted therapies, such as JAK inhibitors and anticytokine mAbs, have revolutionized treatment by selectively modulating pathogenic pathways. For example, TNF‐α inhibitors (e.g., adalimumab) and IL‐17 blockers (e.g., secukinumab) are effective in treating RA and psoriasis, respectively.

Despite these advancements, challenges persist. Broad‐spectrum immunosuppressants such as glucocorticoids and MTX remain first‐line therapies but have systemic side effects. Emerging cell‐targeted therapies, including CAR‐T cells and Treg adoptive‐transfer, offer promise for refractory cases but require further validation in clinical trials. For example, CD19‐targeted CAR‐T therapy has induced remission in SLE by depleting autoreactive B cells. However, long‐term safety and efficacy data are limited, and personalized approaches are needed to address disease heterogeneity.

Future research should focus on identifying biomarkers for early diagnosis and stratifying patients based on molecular subtypes. Multiomics technologies and artificial intelligence may enhance precision medicine by predicting treatment responses. Additionally, combination therapies, integrating biologics with small‐molecule inhibitors or microbiota modulation, could maximize effectiveness while minimizing toxicity. Ultimately, bridging translational gaps between preclinical models and clinical practice will be critical to developing curative strategies for autoimmune diseases.

## Author Contributions

Xiaoshuang Song searched the publications and drafted the manuscript. Hantian Liang, Fang Nan, Wenjing Chen, Junyao Li, and Liu He drafted the manuscript. Yiping Cun, Zhenhong Li, and Wei Zhang edited the manuscript. Dunfang Zhang supervised the work and edited the manuscript. All authors have read and approved the final manuscript for publication.

## Conflicts of Interest

The authors declare no conflicts of interest.

## Ethics Statement

The authors have nothing to report.

## Data Availability

Not Applicable.
